# Educational Relative Value Units as a Measure of Academic Productivity: A Systematic Review

**DOI:** 10.7759/cureus.36995

**Published:** 2023-04-01

**Authors:** Gary L Beck Dallaghan, Sarah T Wright, Jennifer Plant, Lavjay Butani, Bruce Z Morgenstern

**Affiliations:** 1 Department of Medical Education, University of Texas at Tyler School of Medicine, Tyler, USA; 2 Health Sciences Library, University of North Carolina at Chapel Hill, Chapel Hill, USA; 3 Department of Pediatrics, University of California Davis School of Medicine, Sacramento, USA; 4 Continuing Professional Development, Roseman University College of Medicine, Las Vegas, USA

**Keywords:** mission-based budget, mission-based management, medical education, evu, educational value units

## Abstract

Introduction: Academic Health Centers (AHCs) have complex, often competing missions. Many have developed mission-based management (MBM) systems to support their clinical and non-clinical missions. There are limited data on MBM use for their educational missions. Our scoping review explored how AHCs employed such systems.

Materials and methods: Arksey and O’Malley’s six-stage framework guided our review. Based on pre-defined criteria, English language articles from PubMed, EMBASE, SCOPUS, and the Healthcare Administration Database published between 2010 and 2020 were loaded into a reference manager. The search included all health professions education schools. Articles were excluded if they were review articles, commentaries, or clearly did not involve funding for education. From the final list of selected articles, data were extracted using a data extraction sheet we developed. Two researchers reviewed each article again to ensure extracted data were reported consistently and with sufficient detail.

Results: Of the 1729 manuscripts identified, 35 met inclusion criteria. Sixteen (46%) contained data in some form but did not have a formal methods section describing the specific approach to data collection and analysis. Moreover, there was marked variability in how educational effort was quantified, what counted as educational effort (educational scholarship versus teaching) and the impacts of such quantification (departmental funding versus individual faculty incentives). None of the studies reported on the impact on faculty promotion. Faculty satisfaction with the system was reported in seven studies (20%) and was generally positive.

Conclusions: A systematic description of how systems were developed to support the educational mission was lacking. Clear goals, methods of development, uniform data on educational productivity and quality, and program evaluation were not defined by most articles. This lack of process clarity presents a challenge, but more importantly an opportunity for academic health centers to unify efforts and continue to further their educational mission.

## Introduction and background

Unarguably, an academic health center (AHC), herein operationally defined as an entity that provides clinical health care and educates future health care professionals [1], is a business, irrespective of its profit-making goals and tax status. Therefore, an AHC must address its missions to identify ongoing expenses and work toward generating a positive margin, those missions being: clinical care, education, service to the institution, and often, but not invariably, biomedical research. Mission-based management (MBM) is one strategy that was developed as a tool to help businesses address their approaches to budgeting while at the same time ensuring a commitment to the various missions of the institution [2]. In the context specifically of AHCs, for the clinical mission, institutions have almost universally adopted the work relative value unit (wRVU) [3] as a measure of productivity. Systems to quantify the other equally valuable missions, especially education, have, unfortunately, lagged considerably. 

In AHCs, education remains an incompletely supported mandate due to increasing costs that have led centers to prioritize clinical revenue generation and pursuit of research grants. This trend has contributed to mission conflict among clinical faculty [4] who face challenges in balancing their passion to teach with the other demands on an academic faculty member. To address this issue, as part of an evolution to an MBM system [2], the concept of an educational relative value unit (eVU), or similar quantifications of education efforts, has been proposed as a first step in protecting, promoting, and incentivizing the educational mission. While the concept of the eVU is inherently attractive and increases attention directed towards the educational mission of an academic unit, on a practical level there is much variation in how the metric is designed, implemented, and utilized by divisions, departments, and health systems. Equally important to explore are the consequences of such a system on individual faculty, the overall educational mission (e.g., its impact on the quality and quantity of teaching) and the fiscal well-being of the unit. 

The purpose of this scoping review is to explore the published literature on MBM systems adopted by health care institutions that have included a metric to acknowledge the educational mission and quantify educational effort. Our specific aims are to provide an overview of: a) the design of eVU systems in health professions education, b) the components included in eVU systems (what gets credited as “education”), c) how eVU systems are applied at an individual educator level (e.g. incentivization versus penalization), and d) the evaluation of their impact on individuals and programs. The MBM approaches to research and service to the institution are beyond the scope of this review. 

## Review

Our procedure for this literature review followed Arksey and O’Malley’s six-stage framework [5]. This framework includes the following steps: 1) identifying the research question(s); 2) identifying relevant studies; 3) selecting studies; 4) extracting data; 5) summarizing and reporting results; and 6) consulting stakeholders to validate findings. Stage 1 (our specific aims) was described in the introduction section above. Stage 6 was a reflexive process exercised by the research team throughout the article selection, screening, and analysis process. Therefore, we will not further elaborate on these two stages.

Stage 2: identifying relevant studies

One of the authors (BZM) conducted a preliminary search of the literature. We provided this search along with key terms to a medical librarian (STW) who then conducted an extensive literature search. The literature search and review was conducted using Preferred Reporting Items for Systematic reviews and Meta-Analyses (PRISMA) guidelines. Search strategies for each database are provided in Table 1.

**Table 1:** Database Search Strategies

Database	Results	Search Terms
PubMed	1315	(teaching[mesh] OR "medical education"[tiab] OR education, medical[mesh] OR faculty[mesh] OR faculty[tiab] OR Teaching[tiab] OR academic medical centers[mesh] OR "academic medical center"[tiab] OR "academic medical centers"[tiab]) AND (mission-based OR "relative value" OR value-based OR "value unit" OR "Value units" OR rvu[tiab] OR rvus[tiab] OR eVU[tiab] OR eVUs[tiab] OR rbrv[tiab] OR rbrvs[tiab] OR arvu[tiab] OR arvus[tiab] OR "funds flow" OR incentive*[tiab] OR relative value scales[mesh]) NOT (letter[pt] OR comment[pt] OR editorial[pt]) AND ("2010"[Date - Publication] : "3000"[Date - Publication])
Embase	549	(('medical education' OR faculty OR teaching OR 'academic medical center*' OR 'university hospital*' OR education*) NEAR/7 ('mission based' OR 'relative value' OR 'value based' OR 'value unit' OR 'value units' OR rvu OR rvus OR eVU OR eVUs OR rbrv OR rbrvs OR arvu OR arvus OR 'funds flow' OR incentive* OR 'relative value scales')) AND (2010:py OR 2011:py OR 2012:py OR 2013:py OR 2014:py OR 2015:py OR 2016:py OR 2017:py OR 2018:py OR 2019:py OR 2020:py) AND ('article'/it OR 'article in press'/it OR 'review'/it)
SCOPUS	833	TITLE-ABS-KEY ( "medical education" OR "academic medical center*" OR "university hospital*" OR "teaching hospital*" OR ( ( education* OR faculty OR teach* OR academic ) AND ( medical OR hospital ) ) ) W/3 ( "mission based" OR "relative value" OR "value based" OR "value unit" OR "value units" OR rvu OR rvus OR eVU OR eVUs OR rbrv OR rbrvs OR arvu OR arvus OR "funds flow" OR incentive* ) AND ( LIMIT-TO ( DOCTYPE , "ar" ) OR LIMIT-TO ( DOCTYPE , "re" ) )
Healthcare Administration Database	536	(("medical education" OR "academic medical center*" OR "university hospital*" OR "teaching hospital*" OR ((education* OR faculty OR teach* OR academic) AND (medical OR hospital))) AND ("mission based" OR "relative value" OR "value based" OR "value unit" OR "value units" OR rvu OR rvus OR eVU OR eVUs OR rbrv OR rbrvs OR arvu OR arvus OR "funds flow" OR incentive*)) (filter 2010 to present and scholarly journals)

The databases used for this search included: PubMed, EMBASE, SCOPUS, and Healthcare Administration Database. The search included English-language articles published between 2010 and 2020. The search results were loaded into a systematic review reference manager (Covidence®, Melbourne, Australia) to screen out duplicate articles. During the full-text screen and data extraction phase, reference lists were also inspected to identify additional articles for inclusion in the study, and citations of the extracted papers were also searched. The search included studies from all schools/systems involved in any health professions education such as dentistry, nursing and pharmacy.

Stage 3: selecting studies

Each abstract was reviewed by two members of the research team for inclusion or exclusion. If there were disagreements, a third researcher reviewed the abstract to make a final determination. Abstracts were included if they indicated the article involved a discussion about eVUs or a similar metric. Abstracts were excluded if they were from review articles, commentaries, or clearly did not involve funding for education.

After the abstract review process, all selected papers underwent full-text reviews. Each article was reviewed by two members of the research team. Any disagreements were resolved by a third member of the team reviewing the article and making a final determination. Once this process was completed, data were extracted.

Stage 4: extracting data

One of the researchers (BZM) developed a draft data extraction form. The group reviewed, discussed, and edited the information we hoped to cull from the articles. Upon final approval, a finalized extraction form was set up in Covidence. We did not include faculty perception on the development of the eVU rubrics amongst data to be abstracted. We also chose to distinguish between educational scholarship (work aimed at dissemination of a peer-reviewed educational product) from all other educational activities such as teaching, mentoring, assessing learners and curriculum development. This distinction was informed by the models of Boyer [6] and Glassick [7], and was driven partly by the fact that some of the selected papers included “scholarship” with educational activity in their eVU rubrics, whereas other papers separated the two. We divided the final list of articles to extract data from such that each team member was responsible for 10-13 articles. 

Once data had been extracted from each article, the information was exported to an Excel spreadsheet (Microsoft, Redmond, WA, USA) for further summarization and analysis. The final step in the extraction process was for two of the researchers (BZM, GBD) to review each article again to ensure extracted data were reported consistently and with sufficient detail to summarize. The complete table for data extraction is shown in the appendix.

Results

**Figure 1:**
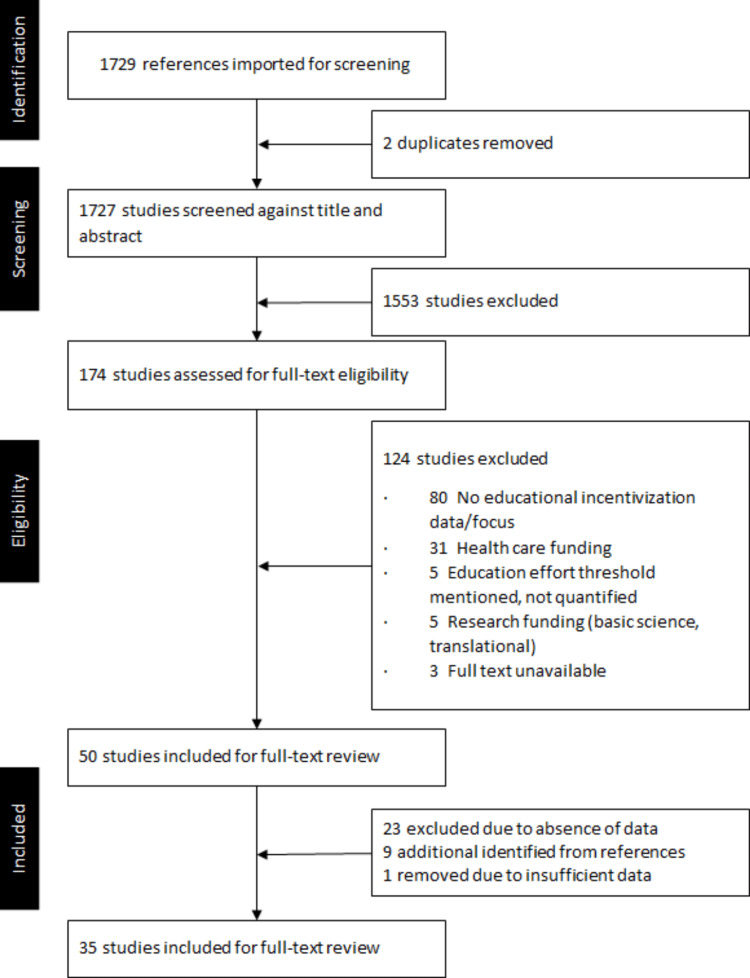
Article selection flow diagram

A total of 1729 papers were identified using the search criteria and subject to the first level of review (titles and abstracts). Of the 50 studies selected for full text review, 23 were excluded due to the absence of data or the lack of a specific eVU plan/implementation. These articles were more of a commentary about the need for such a measurement. This resulted in 27 papers ultimately chosen for review. An additional nine articles were identified from mining the references of these 27 manuscripts. Finally, after final review and abstraction, one more article was removed due to the lack of sufficient data, resulting in a total of 35 papers for inclusion (Figure 1). None of the studies involving schools/systems from non-physician professions made the final cut based on our pre-set inclusion criteria.

Of note, 16 of the 35 papers (46%) contained data in some form but did not have a formal methods section describing the specific approach to data collection and analysis. The abstracted data are presented in Table 2. There is wide variation in the designs of the eVU systems reported. 

**Table 2:** Abstracted data elements from the 35 references - = Not reported; OSCE = objective structured clinical examination; PBL = problem-based learning; 1 PD = program director – student, resident and/or fellowship programs; 2 teaching awards = awards and excellent evaluations from students; 3 QI = any quality improvement activity

Paper	New Lecture	Repeat Lecture	Hospital Teaching: Residents	Hospital Teaching: Residents & Students	Outpt Teaching: Residents	Outpt Teaching: Residents & Students	Value for Mentoring	Other elements
Guiot [8]	Y	Y	Y	Y	Y	Y	Y	Modules, workshops, morning report, ed confs, interviews, advising
Adams [9]	Y	Y	-	-	-	-	-	
Burns [10]	N	N	Y	Y	N	N	Only trainee QI projects	Papers and grants
LeMaire [11]	Y	-	-	-	-	-	Y	PD^1^, teaching awards, excellent evals from learners, oral examiner, “perfect” scores (feedback, observed H&P, submission of evals)
Denton [12]	Y	-	-	-	-	Y	-	OSCES, small-group facilitator
Savides [13]	-	-	Y	-	Y	-	-	Attend Grand Rounds
Backeris [14]	-	-	Y	Y	-	-	-	
Regan [15]	Y	Y	Y	Y	Y	Y	-	Labs, sim, OSCEs, Resident confs, journal club, orientation
Clyburn [16]	Y	Y	N	N	N	N		Sm grp teaching, attend Grand Rounds, phys diagnosis, Foundations of clin med course, interviews
Sakai [17]	Y	Y	Y	Y	Y	Y	Y	PD, school committees, admissions, PBL facilitator, teaching scores, advisor, examiner, program committees
Cramer [18]	Y	Y	Y	Y	Y	Y	Y	Committees, small-group facilitator, PD, new courses, teaching awards^2^, QI^3^
Willis [19]	Y	Y	Y	Y	Y	Y	Y	Sm grp teaching, procedure/lab, advising
Hilton [20]	Y	Y	Y	Y	Y	Y	-	Morning report, committees
Rouan [21]	Y	Y	Y	Y	Y	Y	-	Phys Dx, morning report
Pugh [22]	-	-	-	-	-	-	-	Eval completion, attend conf
Kairouz [23]	Y	Y	Y	Y	Y	Y	-	PD, committees
Mezrich [24]	-	-	N	N	N	N	-	% of effort in classroom teaching ( times “academic value:” 0.3 for residents, 0.2 students); students’ score s of faculty
Bardes [25]	Y	Y	Y	-	Y	-	-	PD, tutor, QI, committees
Yeh [26]	Y	Y	Y	-	Y	-	-	PD, morning report, didactics, interviews
Khan [27]	Y	Y	Y	Y	Y	Y	Y	Workshops, interviews, teaching awards
Martinez [28]	Y	Y	Y	Y	Y	Y	Y	Clin comp committee, scholarly oversight cte, interviews, personal faculty development in med ed, accreditation activities
Carmody [29]	Y	Y	-	-	-	-	Y	PD, QI, accreditation, labs, simulation, didactics attendance, committees, interviews
Leverence [30]	-	-	-	-	-	-	-	Teaching awards
Ma [31]	Y	Y	-	-	-	-	-	Tutoring; committees
House [32]	Y	Y	-	-	-	-	Y	Attendance at confs, committees, eval completion, interviews
Morrow [33]	Y	Y	-	-	-	-	-	PD
Ridley [6]	-	-	-	-	-	-	Y	PD, committees
Stites [34]	Y	Y	Y	Y	Y	Y	-	PD,
Reece [35]	Y	Y	Y	Y	Y	Y	Y	Teaching hours, teaching awards, PD, conference attendance
Filler [36]	-	-	-	-	-	-	-	Teaching awards, PD, levels of performance
Sloan [37]	Y	Y	-	Y	-	Y	-	
Williams [38]	Y	Y	Y	Y	-	-	Y	PD, Organizing conferences, small-groups, exam writing, teaching awards
Howell [39]	-	-	-	-	-	-	-	
Anders [40]	-	-	-	-	-	-	-	
Hales [41]	-	-	Y	Y	Y	Y	Y	PD

Settings and Size of Studies/Reports

Three of the studies (9%) reported on a (medical) school-wide roll out of their system. Two reports (6%) were focused on a hospital setting - in both instances a free-standing children’s hospital. The remaining 85% were limited to a single department, although some of the departments were quite large and included specialty divisions (Table 3). The numbers of faculty ranged from 11 to 893; although in eight reports (23%), specific numbers were not reported. 

**Table 3:** Settings of the program roll-out in the included reports *Total exceeds 100% due to rounding; ^1^ University of Pittsburgh Medical Center; ^2^ University of California, Davis

Site	N (%)*	Comment
School-wide	3 (9)	
Hospital-wide	2 (6)	Both children’s hospitals
Department		
Internal Medicine (IM)	8 (23)	One was survey of IM Depts
Emergency Dept (ED)	6 (17)	One Peds ED
Pathology	3 (9)	
Anesthesia	3 (9)	Same program^1^ – different systems
Family Medicine	2 (6)	
Psychiatry	2 (6)	Same program^2^ – updated report
Surgery	2 (6)	
Gastroenterology	1 (3)	
Primary Care	1 (3)	
Radiology	1 (3)	
Pediatrics	1 (3)	

Educational Productivity Change

As noted earlier, we chose to separate teaching and educational productivity from educational scholarship in our data abstraction of the papers. Eighteen of 35 (49%) studies included in the review reported data on educational productivity, as defined by us (Table 4). An increase in productivity was reported in 12, no change was reported in three, a decrease was noted in one, and in two reports, productivity increased for some faculty and decreased for others. Scholarly activities, including those related to education, were often not separated from educational activities as we have defined them; the rubrics included scholarship as an eVU-generating activity. In the 11 papers that report increases in educational productivity, the increase was, using our construct, often in the realm of scholarship [6,10,17,29,30]. The papers do not report if the increased scholarly output was in the domain of clinical practice, educational scholarship, or scientific research.

**Table 4:** Impact of an eVU/Mission-based tracking system on “educational productivity” UPMC=University of Pittsburgh Medical Center, eVU=educational relative value unit

Paper	Impact on ed productivity	Comments
Adams [9]	More lectures given; more presenters asked back	UPMC 2^nd^ report.
LeMaire [11]	More scholarship	Increased presentations, publications, grant funding, clinical trials, committee positions, editorial board positions
Burns [10]	Increased	Scholarship productivity exceeded that for educational activities
Regan [15]	Increased for some, reduced for others	1^st^ year of program. Three/47 earned more eVU than anticipated, 34 earned fewer (felt to be departmental overestimate); 6 failed to complete assigned eVU obligations
Clyburn [16]	Increased for some, reduced for others	Increase: Biostatistics, Cardiology, Emergency Med, GI, General internal Med, Hematology/Oncology, Infectious Disease, Pulmonary Decrease: Endocrinology, Nephrology, Rheumatology
Sakai [17]	Increased matrix points for 36% (Yr 1), 33% (Yr 2), 28% (Yr 3), and 25% (Yr 4)	UPMC 2^nd^ report; Providers had to earn merit matrix points. Primary endpoint for academic productivity was peer-reviewed publications, which increased annually.
Cramer [18]	Mean points for teaching: year 1 = 1194 year 2 = 1032 year 3 = 1146	No statistical analyses applied to eVU data; ranges suggest these numbers did not statistically differ. “Scholarship” – grants, peer-reviewed papers, presentations – quantified separately. Scholarship of teaching and learning not separated out.
Rouan [21]	Dollars follow teaching activity. Increased for some, reduced for others	Increase over baseline: Cardio, GI, ID, Immunology, Nephrology, Pulmonary Decrease: Endocrinology, Hematology/Oncology Money to General Internal Med stable – directly from medical school. Unclear impact in that division of program.
Pugh [22]	Administrative measures improved	90-day resident evaluation completion rate 71.8% ®100%. Mean conference attendance unchanged.
Khan [27]	Increased	From year 1 and 3, mean group educational productivity increased from 73% to 88% of expected, and mean individual productivity increased from 54% to 82% of expected.
Carmody [29]	Administrative measures improved	Conference attendance increased 21%; the number of resident assessments completed increased by 30%. 1240 academic activities logged in new system – no baseline data to compare.
Carmody [29]	Increased hours	Teaching hours increased by 8% over 3 years – not statistically different. Total publications did increase statistically significantly. Incentive dollars increased from a mean of $3,191 to $11,153.
Leverence [30]	Median academic bonus fairly constant over 10 years	Scholarship of teaching and learning not separated from scholarship points. Total academic bonus rose linearly among faculty in the bottom three quartiles of academic productivity; increased exponentially for those in the 75^th^ to 100^th^ percentile.
Ma [31]	Administrative measures improved	Total eVUs increased from 94.4 pre-implementation to 109.8 post. Conference attendance eVUs increased from 22.7 to 34.5. eVUs for evaluation completion rose from 5.9 to 8.8 – all 3 of these measures increased statistically significantly.
House [32]	Increased	Preliminary report; no specific data. “There has been evidence of increased academic productivity at both the department level and the individual faculty level.”
Ridley [6]	Increased	Not quantified: faculty participation in resident teaching and attendance at conferences “dramatically increased.” Scheduling faculty for lectures became easier: faculty attendance at resident morning report improved.
Stites [34]	Academic productivity units remained stable	2-year follow-up. Those more clinically productive were also more academically productive. No difference between junior and senior faculty.
Williams [38]	Scores remained stable	Mean overall scores, as well as in domains of clinical practice, education, scholarship, and administration scores did not change significantly. Overall scores for assistant professors did increase: not reported in which domain.
Filler [36]	Scores remained stable	Mean overall scores, as well as in domains of clinical practice, education, scholarship, and administration scores did not change significantly. Overall scores for assistant professors did increase: not reported in which domain.

Department Funds Flow

Six of 35 papers (17%) reported data on change in flow of funds to departments and/or divisions. In two studies [24,30], department funds either increased or decreased, depending on performance of the overall department/division. For the other four [23,29,31,32], funds flow to departments/divisions increased.

Departments (and hospitals) reported that they had the support of the leadership to begin the process. Traditional budgets had to evolve to accommodate the new systems. Revenues typically were a mix of allocations from the school, the practice plan, and other sources of income. Increased funds as a result of eVU systems were typically the result of reallocation of university dollars in support of mission-necessary faculty efforts (e.g., “teaching activities”), as well as increased clinical revenues and grant support. 

Provider Funds Flow

Twelve of 35 papers (34%) reported data on changes in flow of funds to individual faculty. In nine (75%) of these 12, faculty were eligible to receive incentive dollars. In two (17%) of these 12, individual salaries went up or went down [19,34]. If the salary dropped, this was due to a decrement in university support based on less than anticipated/expected educational effort; decrements were typically offset by increased clinical revenue. In one paper [10], incentive dollars were collected by eligible faculty, but the data demonstrate the bonus was for “scholarship” (e.g., papers and grants) as opposed to “teaching.” It is not clear if any of these scholarship increments were based on productivity in the scholarship of teaching and learning. 

In five additional papers (14%), it is not clear how bonus dollars were distributed, even if faculty met or exceeded their personal targets. These five papers will be discussed individually. For instance, Rouan et al. reported that redistribution of teaching dollars among divisions increased 11.4% [21]. Based on the description of their plan, readers were left to presume funding was distributed to individuals if they met or exceeded thresholds. However, the Division of General Internal Medicine (GIM) was compensated using a rubric that differed from the other divisions and faculty did receive incentive monies. How other divisions in the department distributed funds was not clearly articulated. 

In the second of the five papers, Pugh et al. reported that providers who achieved both of the pre-set benchmarks for evaluation completion and attendance were to be monetarily incentivized each quarter [22]. Faculty significantly achieved/exceeded expectations for percent completion of resident evaluations and did so in a shorter time frame. There was no significant increase in the average faculty attendance at educational sessions. The paper does not explicitly state if distribution of incentives indeed occurred and if so, how many faculty received the incentive. 

In year three of the rollout of a third program [27], 12 of 22 providers met or exceeded quotas and, per protocol, should have received incentive payments; this is not explicitly stated. In the fourth program [29], there were providers who met criteria for incentives, but the amounts had not yet been determined at the time of publication. Finally, in the fifth paper, Filler et al. reported assistant professors had greater improvement in a self-scored ‘scholarship score’ (comprised of various components for scholarship in the domains of clinical practice, education, research, and administration) than did associate or full professors [36]. This then translated into an incentive payment, although neither individual nor mean data are reported.

Scholarly Activity

Twenty of the studies (57%) reported on how scholarly activity was accounted for as part of the mission-based process (Table 5). Typically, the reports identified high-level categories as grant revenue, peer-reviewed publications, and services like editor of a journal or membership on a study section. As noted earlier in the section on educational productivity results, it was rare for the papers to clearly delineate if the scholarship was centered around discovery research (clinical, basic, and/or translational), clinical work (e.g., quality improvement) or the science of teaching and learning. 

**Table 5:** Scholarly activity included in rubrics for eVUs *A more complete abstraction of the research elements of these programs is beyond the scope of this paper. eVU=educational relative value unit

Paper	*Clinical Research (Grants)	*Clinical Research (Papers)	*Bench Research (Grants)	*Bench Research (Papers)	Educational Scholarship (Grants)	Educational Scholarship (Papers)	Educational Scholarship (Presentations)	Other Elements
Guiot [8]	-	-	-	-	-	-	Y	
Burns [10]	Y	Y	Y	Y	Y	Y	Only Jr & Mid-career	
LeMaire [11]	Y	Y	Y	Y	Y	Y	Y	Leadership, Peer-review, innovation
Savides [13]	Y	Y	Y	Y	Y	Y	Y	
Clyburn [16]	-	-	-	-	-	-	-	Separate process
Sakai [17]	Y	Y	Y	Y	Y	Y	Y	Editor, peer-review
Cramer [18]	Y	Y	Y	Y	Y	Y	Y	Editor, peer-review
Willis [19]	Y	Y	Y	Y	Y	Y	Y	
Hilton [20]	Y	Y	Y	Y	Y	Y	Y	
Kairouz [23]	Y	Y	Y	Y	Y	Y		
Mezrich [24]	Y	Y	Y	Y	Y	Y	Y	
Khan [27]	-	-	-	-	-	-	-	Book chapters, peer review, editorial board
Martinez [28]	-	-	-	-	-	-	-	Project with resident, paper with resident
Carmody [29]	Y	Y	Y	Y	Y	Y	Y	Chapter, journal editor, peer review
Leverence [30]	Y	-	Y	-	Y	-	-	
Ma [31]	Y	Y	Y	Y	Y	Y	Y	Editorial activity, study section
Morrow [33]	Y	-	Y	-	Y	-	-	
Ridley [6]	Y		Y		Y			
Stites [34]	Y	Y	Y	Y	Y	Y	Y	
Reece [35]	Y	Y	Y	Y	Y	Y	Y	Editorial activity
Filler [36]	Y	Y	Y	Y	Y	Y	Y	
Sloan [37]	Y	Y	Y	Y	Y	Y	Y	
Williams [38]	Y	Y	Y	Y	Y	Y	-	Editorial activity
Hales [41]								

Promotion and Tenure

None of the studies reported specific data related to the impact of eVU systems on faculty promotions. One report noted that meeting eVUs was considered as part of the promotion application [27]. Reports that included the comprehensive mission-based programs, ones that addressed all three to four components of a clinical faculty member’s job - clinical care, education, scholarship, service - did demonstrate an increase in grants and papers, which presumably correlate with successful professional advancement and promotion [18].

Clinical Productivity

Six of the 35 papers (17%) reported on the impact of implementation on clinical productivity. Clinical productivity increased, with two of these six papers reporting in more detail; in one [36], clinical productivity only increased for assistant professors and was stable for the others. In the other paper [18], clinical productivity increased for some faculty and decreased for others.

Faculty Perspectives

Seven reports (20%) contained data, generally positive, on the satisfaction of the faculty after the program was implemented. 

Impact on Learners/Learner Perspectives on Faculty Performance After Roll Out of an eVU System

Three of the papers (9%) included a pre-post implementation evaluation of learner perspectives on teaching by faculty participating in the eVU program. Two of these were from the University of Pittsburgh Medical Center (UPMC). In the first [14], the eVU system in part used teaching ratings by learners as a method determine part of faculty eVUs; no change in ratings was evident. For the second [9] the eVU rubric was different than the first UPMC report. On average, there were better learner scores of teaching sessions, and more presenters were requested by the learners to return for subsequent sessions. 

The third report that contained learners’ perspectives was from the University of Queensland/Ochsner (UQ-OCS) general practice clerkship (GPC) [12]. The authors simply report, without further elaboration, that the GPC was “the top-rated third-year clerkship at the UQ-OCS for the first three years of clinical rotations at the school.” The authors did not report that their ranking was a result of support through their eVU system.

Discussion

Around 25 years ago, MBM proponents began to develop the concept of the eVU [4,25] as one way to level the playing field by highlighting the educational mission of an AHC and quantifying it to a level similar to the measurement of the clinical mission product via wRVUs. We undertook this scoping review to explore the published literature on MBM/eVU systems adopted by AHCs that have included specific metrics to acknowledge the educational mission and to quantify educational effort. The 35 papers that were reviewed and abstracted for inclusion in this scoping review were heterogenous in their scope and purpose. Many, it seemed, were published as proof of concept papers, demonstrating that an MBM approach to the education mission was possible, albeit complex. Others demonstrated some improvements in specific aspects of an educational program (e.g., completion of learner evaluations [22]) associated with some effort to incentivize the desired behaviors. The nature of the papers was such that often there was not a clear methods section, so we had to infer that certain measures were made, even if they were not described. For example, authors may have stated that faculty were “accepting of” or “satisfied with” an eVU program that had been initiated, although how the authors arrived at those conclusions was not clear. As we abstracted the manuscripts, we accepted that, for the program described in the paper, faculty acceptance/satisfaction was measured. 

Recommendations for Future eVU Studies/Reports

Having reviewed and summarized the extant eVU data at a granular level, we now wish to step back and reframe the discussion with a goal to identifying paths to make future studies on eVU systems implementation more consistent and useful. What is clear from this review is that the concept of an eVU-based MBM approach to measuring educational activity is possible, although the components of such systems are complex to develop and, in some cases, to measure. Accordingly, we have identified aspects that we feel should be addressed in future studies of such systems, to facilitate adoption by other institutions:

Comprehensive approach: Ideally, a study of an eVU program should be as comprehensive as possible, addressing all the aspects of the educational program. Studies should have specific aims identified in the manuscripts, especially if only one or two aspects of the eVU program are to be investigated. Methods specific to those aims need to be clearly described. 

Address all aspects of a faculty member’s roles: The roll-out of an eVU program may have unanticipated effects on the other aspects of a clinician-educator’s efforts and productivity, so while the description of an eVU program will, by definition, address the educational activity of faculty, the measures should also address the impact on faculty’s clinical productivity, scholarly productivity, and service activity. 

Broadly address scholarship: The faculty who are teaching students and residents are largely doing so in clinical realms. Scholarship for such faculty needs to be addressed broadly, such as in the framework proposed by Boyer (Discovery, Application, Integration, Teaching/Learning) [42]. As noted in Table 5, we assumed that if scholarly productivity was measured, at least in terms of grants, papers and presentations, the scholarship of teaching and learning and other educational pursuits would have been captured, but none of the papers reviewed specifically identified how scholarship writ large, as in the Boyer framework [43], was identified, and our assumption may not be correct. 

Broadly address educational effort: The educational activities of the clinician-educator are many and eVU programs that aim to measure educational activity should make the effort to capture all aspects, including, but not limited to delivering and/or planning lectures, leading active learning/flipped classroom sessions, developing curricula, assessing learners, attending conferences, offering feedback, completing required documentation (grades, mid-course evaluations), collaborating on interdisciplinary and interprofessional efforts, bedside teaching, etc. The Centers for Medicare and Medicaid Services [44], especially through their work with the American Medical Association’s RVS Update Committee [45], have simplified the task of applying very specific wRVU values to procedures. In contrast, there are limited extant lists of the many educational activities that should be assigned eVU values [42]. Although not published, the University of Virginia School of Medicine has a detailed plan for their eVU structure [46]. It is important for those institutions with clear descriptions and how they were derived to make them public for others to adapt. 

Address quality of educational activity: Optimally, although for this there is no simple solution, some measure of quality associated with these efforts should be a component of a system that purports to measure educational “value.” As examples, prompt but perfunctory completion of learner evaluations without useful feedback for the learners and thoughtful narratives for the program directors is of minimal use both to the program and the learners. Repetitive offering of a lecture without incorporating prior feedback, may reflect “activity,” but not activity of value to the AHC, and certainly not value to the learner. The tools to achieve measures of such quality require development and validation but must not be overlooked for the sake of simple, objective process measures (e.g., attendance at conferences or timely completion of evaluations).

Address faculty perspectives: It will be critical to obtain well-defined and well-developed assessments of faculty perspectives as any eVU system is developed and implemented. At a minimum, we believe that faculty should be surveyed via some means for their perceptions on the processes used in the development of the rubrics that underlie the eVU program, of the “final rubric” that is adopted, of the roll-out of the system and processes implemented to collect data, of their satisfaction with the flexibility and adaptability of the program, and of the impact of the program on their job satisfaction. In parallel, as programs mature, data on the promotion and advancement of faculty need to be collected and analyzed.

Address impact on learners and the learners’ perspectives: Finally, while the concept of an eVU-like MBM program is, at its root, something in place to have manageable metrics of “productivity [46],” we cannot lose sight of the reality that for an AHC, specifically for its educational mission, learners are the main customers/stakeholders. Evaluation of the impact of an eVU system on learners is also quite critical. Measures could include performance data on written and competence exams, pre-post (if possible) assessments of the faculty’s performance from the student perspective, and of course, student acceptance/reaction to faculty. In theory, faculty who know that they are expected to help learners learn, and who know that these expectations are built into their annual performance targets may be more inclined to focus more effort and energy on their teaching demands.

## Conclusions

Based on the findings from this scoping review, we anticipated that these data would provide insights for institutions considering adopting an eVU metric and potentially enable current eVU users to refine their processes. However, we only found a small number of studies with published concrete outcomes data. The lack of clarity surrounding these processes presents a challenge to institutions that they carefully study their MBB/eVU systems and report their process and outcomes for others to learn.

## Appendices

**Table 6:** Supplemental Data: Data extraction information for the articles used in this review UPMC=University of Pittsburgh Medical Center, UCSD=University of California San Diego, OHSU=Oregon Health and Science University; eVU=educational relative value unit, RVU=relative value unit, wRVU=work relative value unit, TFTE=teaching full-time equivalent

Study	Study Methods	Focus of Study	Number of Departments (if applicable)	Number of Faculty	Primary Outcome	Faculty Acceptance/Satisfaction Measured	Clinical Productivity Change	Educational Productivity Change	Learner Perspective of EVU Measured	Funds Flow Change to Department	Funds Flow Change to Provider	Impact on Promotion	Value per Lecture (NEW)	Value per Lecture (REPEAT)	Value for Clinical Teaching (Hospital Team - Residents)	Value for Clinical Teaching (Hospital Team + Students)	Value for Clinical Teaching (Clinic Residents)	Value for Clinical Teaching (Clinic + Students)	Value for Clinical Research (Grants)	Value for Clinical Research (Papers)	Value for Bench Research (Grants)	Value for Bench Research (Papers)	Value for Educational Scholarship (Grants)	Value for Educational Scholarship (Papers)	Value for Educational Scholarship (Presentations)	Value for Mentoring	Other Elements Assigned Value	Additional Notes (Optional)	Useful?
543: Educational Added Value Unit: Development and Testing of a Measure for Educational Activities, Guiot, Hospital Pediatrics, 2017 [8]	Survey - data handled quant	Children's Hospital	5/23 divisions	74/119	Faculty acceptance; Total units earned by role	No	N/A	N/A - 1 year/ no baseline	No	N/A	N/A	N/A	0.8 "points" - see table 1	0.5 "points"	1 "point"	1 "point"	1 "point"	1 "point"	N/A	N/A	N/A	N/A	N/A	N/A	0.8 "points"	1 "point"	e-learning module, workshop, morning conference, ed committees, interviews, advising		For table 1, perhaps. No real impact data. Table 1 is uploaded to the shared folder
231: Financial Incentive, in Place of Nonclinical Time, Increases Faculty Involvement and Improves Resident Didactic Evaluation Scores in an Anesthesiology Residency Training Program, Adams, J of Education on Perioperative Medicine, 2019 [9]	Quantitative	Department	Anesthesiology - UPMC	63/220	Number of faculty presenting lectures; secondary measure- learner assessment of lecture quality	No	No	Yes	Yes - pre-post design. Lectures rated higher, presenter more likely to be wanted back	No	Yes - $500 for presenting a lecture. Total # of lectures didn't change, but more staff gave them	No	$500	$500	No	No	No	No	No	No	No	No	No	No	No	No	Classroom teaching		Limited. Dept decided to incentivize resident didactic series by paying attendings, rather than with time off. Residents thought the presentations were better.
400: The Evolution of Earned..., Burns, Academic Pathology, 2018 [10]	Other: Descriptive study on development of incentive plan- eVU, research value, QI value (and some support in a different mechanism to educational leadership/administration) BZM thinks quant	Department	Pathology	97	Description of change in what was rewarded and also gender bias, pre and post implementation of a new system for incentivization	No	NR	NR	No	NA	Sort of - not clearly defined as to how points coverted to $ Part C of plan. Points assigned. "Education" pts no Δ, Papers & funding ↑	NR	No	No	No	No	No	No	Yes-grants	Yes	Yes	Yes	Yes	Yes	Only for junior/mid career faculty	Only for mentoring a trainee QI project	QA and QI work (see table 6) and supporting diversity		More than most. I can't determine their final step - academic points into $.
338: An Academic Relative Value Unit System for Incentivizing the Academic Productivity of Surgery Faculty Members, LeMaire SA, Ann Surg, 2018 [11]	Quantitative	Department(s)	Surgery - 11 divisions	142	Development of academic RVU compensation program	No	NR - There were more $, but from dept budget and/or surplus collections	Increased number of presentations but similar for publications (Table 4 has breakdown of increases) Not in classical education activities	NR	Compensation through dept compensation plan, which is part university $ and part clinical revenue	$10K if in top 10%, $5 if top 33%, $2500 if in top 50%. Raw aVRU's multiplied by 3 for Asst Prof, 2 for Assoc Prof. 50% of Dept surplus allocated to aRVU comp program Since 2015, $493,900 in aRVU bonuses were awarded to 59 faculty members - 58% of eligible faculty.	NR	Teaching (clinical or class) activities scaled: 175-375	NR	NR	NR	NR	NR	100-625 (not specific to clinical, basic sci, ed)	125-750 (scaled by impact factor)	200-1250 points	125-750	? 200-1250	? 125-750	25-150	175-375	Leadership, Scientific peer review, innovation	Points were developed for academic activities.	Yes
908: Recruiting Primary Care Physicians, Denton, Academic Medicine, 2015 [12]	Quantitative	Summary of EVU system for clinical educators in a primary care department (family medicine & internal medicine) at Ochsner Clinical School	2	44 in 2012, 48 in 2013, 65 in 2014	Recruitment of clinic teaching faculty by buying out clinical time	Not reported	Not reported	no	Overall impression of the clerkship is highly rated	Yes	Historical clinical productivity data showed that the average productivity during 40 minutes was 2.5 RVUs. One EVU was given a value equal to that of one primary care RVU or $42. Accordingly, time spent teaching a student during a half-day clinic session would be worth 2.5 EVUs or $105. No data in $ to providers	Not reported	Yes	Not specified	Not reported	Not reported	Not reported	Yes	Not reported	Not reported	Not reported	Not reported	Not reported	Not reported	Not reported	Not reported	EVUs are also added for serving as an objective structured clinical examination evaluator, for providing lectures to students, and for serving as a small-group tutor. EVUs are not added for attending faculty development or evaluation sessions. EVUs are given to all faculty who worked with students and do not depend on student ratings, attendance at faculty development conferences, completion of student evaluations, or other academic measures of quality. The purpose of EVU tracking and reimbursement is solely to account for the faculty member’s lost clinical productivity when a student is present.	2012: 2,658 EVUs for 44 PC faculty & 30 students. 2013: 2,872 EVUs for 48 PC faculty & 30 students. Tuition to cover EVUs = $121,573. 2014: 6,414 EVUs for 65 PC faculty & 67 students. Tuition to cover EVUs = $205,632. Shortfall from tuition picked up by Ochsner	Yes
981: Impact of the relative value unit-based system on academic centers, Savides T, Gastrointestinal Endoscopy, 2014 [13]	Quantitative	Gastroenterologist wRVU system at UCSD	1	Not reported	Explanation of wRVU system that builds in compensation for education	Not reported	NR	To incentivize desirable clinical citizenship, teaching excellence, and academic performance, we also withhold 10% of the potential salary that the faculty member can earn based on a 10-point system. No data reported	Not reported	Not reported	Expectations based on past year wRVU, departmental average and # extramural funds No data on funds to providers	Not reported	Not reported	Not reported	Fellow teaching evaluation (1 pt)	Not reported	Fellow teaching evaluation (1 pt)	Not reported	Grant funding (2 pts)	Publish scholarly work: as collaborator (1 pt), as lead or senior author (2 pts)	Same as Clinical Research	Same as Clinical Research	Same as Clinical Research	Same as Clinical Research	National society committee (1 pt); Industry lecture (1 pt); Invited talk at regional/national meeting (2 pts)	Not reported	Table 1 offers breakdown of additional components for the incentive plans		Yes
1083: Impact of a productivity-based compensation system on faculty clinical teaching scores, as evaluated by anesthesiology residents, Backeris J, Clin Anesthesia, 2013 [14]	Survey - data handled quant	Department(s)	Anesthesiology - UPMC	133	The results showed that the Department’s comprehensive performance-based faculty compensation system did not appear to improve overall OR teaching scores among the academic faculty.	No	Not reported	Not reported	To an extent. Their evaluations provide the merit matrix score.	Not reported	Specific merit matrix points are assigned to each academic activity, and the academic faculty earn merit matrix points from any of the three main areas of academic activity: research, education, and administration. Faculty OR teaching scores are one component of the education area. Merit matrix points are assigned to the faculty OR teaching score in the following manner: the average overall score of 7.00 - 8.00 is counted as 50 points, 8.01 - 8.50 as 75 points, and 8.51 - 9.00 as 100 points. One merit matrix point is roughly equal to $100 of salary at risk. In our performance-based faculty compensation system, one full-time equivalent is defined as equal to 2,300 merit matrix points. No data on $ to providers	Not reported	Not reported	Not reported	Yes	Yes	Not reported	Not reported	Not reported	Not reported	Not reported	Not reported	Not reported	Not reported	Not reported	Not reported	Faculty in the clinical track devote 100% of their time to clinical activity. Those in the academic track may spend up to 80% of their time on academic (nonclinical) activity. In both tracks, around 30% of the total annual compensation is productivity based. While clinical faculty also receive OR teaching scores, it is important to note that those scores do not result in financial compensation. Their salary is linked solely to clinical productivity.		Yes
1227: Educational Value Units: A Mission-Based Approach to Assigning and Monitoring Faculty Teaching Activities in an Academic Medical Department, Regan, Academic Medicine, 2016 [15]	Quantitative	Department(s)	Emergency Medicine	54	The teaching grid distributed 5,896 EVUs among 54 faculty members for academic year 2013–2014 (Table 1). Assigned EVU obligations included 3,942 EVUs for educational contact time activities and 1,954 EVUs for administrative activities related to education (see above). Of the participating faculty members, 26 were full-time faculty, 10 were full-time faculty in education-focused career paths, and 18 were part-time faculty or fellows.	No	Not reported	At the end of academic year 2013–2014, complete EVU data were available for 47 faculty members; 7 faculty members did not complete the year for various reasons. Of the faculty that did complete the year, 3 faculty members, all in educational leadership, expended effort greater than 10% of their assigned EVU obligations. However, this burden was significantly less than what these faculty members had experienced prior to the EVU system, demonstrating a more equitable distribution of teaching efforts among faculty. Educational efforts were distributed more equitably across faculty, with less undue burden falling on educational leaders and more widespread faculty participation.	No	Not reported	The department underwrote 5% of the base salary for full-time faculty members (assuming they met their minimum of 100 EVUs) and paid this amount as a supplement. 1 year data, no report re funds to providers	Not reported	1 EVU for each hour of contact time (no credit for preparation) for student and resident activities (not fellows)	Same as above	1 EVU for each hour of contact time	1 EVU for each hour of contact time	1 EVU for each hour of contact time	1 EVU for each hour of contact time	N/A	N/A	N/A	N/A	None	None	None	N/A		We determined that to meet our core educational mission, all full-time faculty should earn a minimum of 100 EVUs annually (5% of annual effort), and full-time faculty on education-focused career paths should earn a minimum 400 EVUs (20% of annual effort). We also felt that part-time faculty and fellows should have obligations concordant with the definition of faculty,1 and thus we required a minimum 50 EVUs annually (2.5% of annual effort) for this group. To underscore the importance of education to our mission, the department underwrote 5% of the base salary for full-time faculty members (assuming they met their minimum of 100 EVUs) and paid this amount as a supplement. By formally allocating financial resources to our educational mission, we sought to reframe faculty perceptions of teaching, so that it was seen as an integral component of the scholarly expectations of all faculty members, rather than as volunteer work.	Yes
1454: Valuing the Education Mission, Clyburn, The American Journal of Medicine, 2011 [16]	Quantitative	Department	1 (IM), with 11 divisions	289	Eight of our 11 divisions increased in faculty size; 2 divisions remained unchanged, general internal medicine and nephrology, and only endocrinology decreased in faculty size. Educational value units increased over time in 8 divisions and decreased in 3 divisions (endocrinology, nephrology, and rheumatology). Clinical productivity increased in all clinical divisions. The formula allocating funds to divisions based on mission resulted in reallocation of more than $500,000 over 4 years. Five divisions saw their budget allocations remain stable, but 3 divisions lost funding: cardiology, gastroenterology, and rheumatology. Three other divisions gained in their allocation: general internal medicine, infectious diseases, and pulmonary.	No	Yes-increased in all divisions	Increased in some divisions, remained stable in the majority, but decreased in others	No	Departmental funds flow to the divisions went up or down	Yes Takes work to interpret. The average faculty member had a drop in their shortfall of revenue when EVUs were counted. Authors note this probably helped avoid salary reductions	Not studied	Yes	Yes	No bedside teaching credit	No bedside teaching credit	No bedside teaching credit	No bedside teaching credit	No	No	No	No	No	No	No	No	Physical Diagnosis Foundations of Clinical Medicine (small group teaching) Medical Student Clerkship Lectures Morning Report Participation Medical Grand Rounds and Grand Rounds Lecturer Noon Conference Lecturer Journal Club/Morbidity and Mortality Facilitator Residency Program Interviewer Program and Clerkship Director, Associate Program Directors Education Committee Member Faculty Development Activities	Clinial and Bedside teaching not included due to them being billable activities anyway. Fellow education was also not included as there are unique attributes about that allowing for billable activities. See jpg image in file for EVU breakdown	YES
1678: Integration of academic and clinical performance-based faculty compensation plans: a system and its impact on an anaesthesiology department, Sakai T, Br J Anaesth, 2013 [17]	Quantitative	Department(s)	Anesthesiology - UPMC	Not reported	This report describes a productivity-based faculty compensation scheme that can enhance clinical and academic productivity.	No	130% Site Mean as target	>150 merit matrix points...declined over course of study	No	Not reported	Not reported	Not reported	Detailed in Appendix 2	Detailed in Appendix 2	Detailed in Appendix 2 (based on resident evaluations of teaching)	Detailed in Appendix 2 (based on student evaluations of teaching)	Not reported	Not reported	Detailed in Appendix 1	Detailed in Appendix 1	Detailed in Appendix 1	Detailed in Appendix 1	Detailed in Appendix 1	Detailed in Appendix 1	Detailed in Appendix 1	Detailed in Appendix 2	Appendix 3	A complete list of academic merit points is in Appendix A, B, & C	Yes
NEW: Implementing a Comprehensive Relative-value-based Incentive Plan in an Academic Family Medicine Department, Cramer, Acad Med, 2000 [18]	Quantitative	Department(s)	Family Medicine	38-49	The first three years of a department-wide incentive plan was described. Increases were noted in teaching and research as well as clinical care.	The authors alluded to the fact that buy-in of the program was not good due to speculation about the accuracy of data as well as delays in reporting from clinical work.	Yes, during year 2 points increased then leveled off in year 3 (less than year 1 benchmark)	Teaching stayed roughly the same across three years.	No	Not reported, after year 1 set-aside	4% salary withheld to provide bonus incentives at the end of the year. Yes, points-based, with a floating conversion factor.	Not reported	Appendix B details breakdown of educational RVUs	No	Appendix A details calculation of clinic precepting	Appendix A details calculation of clinic precepting	Appendix A details calculation of clinic precepting	Appendix A details calculation of clinic precepting	Appendix C details Scholarship RVUs	Appendix C details Scholarship RVUs	Appendix C details Scholarship RVUs	Appendix C details Scholarship RVUs	Appendix C details Scholarship RVUs	Appendix C details Scholarship RVUs	Appendix C details Scholarship RVUs	Not reported	Not reported		Yes
NEW An incentive compensation system that rewards individual and corporate productivity. Willis, Fam Med, 2004 [19]	Quantitative	Description of a comprehensive compensation plan	Family Medicine	18	The explanation of the compensation plan is made that incorporates educational activities in addition to clinic revenue. A satisfaction survey was administered and reported as well, which showed the majority were either neutral or dissatisfied with the system.	Yes	Not specifically reported. It was noted that after implementation, some made significantly less and other more.	Not reported	No	No	Yes, from 2000-2001 one made $3K less and one made $4K more	Not reported	0.75	0.75	4	4	2	2	See table 3	1	See table 3	1	See table 3	1	2.5	2	Table 3 has a lot of information		Yes
NEW: A relative-value-based system for calculating faculty productivity in teaching, research, administration, and patient care, Hilton, Acad Med, 1997 [20]	Quantitative	Described the development of a comprehensive relative-value and time-based system, incorporating aspects of teaching, research, administration, and patient care.	Medicine	17 "super faculty"	The system provided a quantitative method for equal recognition of faculty productivity in multiple areas. One didn't have to excel in all areas in order to be fairly compensated or rewarded for work.	No	Not reported	Not reported	Not reported	Not reported	Not reported	Not reported	3	3	4	4	3	3	2-6	1-2	2-6	1-2	2-6	1-2	1-3	Not reported	Other elements are in Appendix 1		Yes
NEW: Rewarding teaching faculty with a reimbursement plan, Rouan J, Gen Intern Med, 1999 [21]	Quantitative	The purpose was to develop a system for measuring teaching effort and to implement a payment system based on it. Pre and post comparisons of teaching effort were measured.	Internal Medicine	Not reported	There was a change in allocation of teaching dollars to individual divisions after implementation, resulting in a decrease by 28.5% to endocrinology.	Not reported	Not reported	Yes. Redistribution of discretionary teaching dollars among divisions saw an increase of 11.4%. The distribution of teaching units among divisions reflected ABIM and ITE subspecialty question proportions.	Teaching dollars up or down, depending on division	Up or down	To divisions as well as individual providers. Presumably, but individual data not reported, even on average	Not reported	5-10 units depending on lecture type	5-10 units depending on lecture type	70-100 units depending on hospital	70-100 units depending on hospital	25-75 units (based on outpatient visits)	25-75 units (based on outpatient visits)	No	No	No	No	No	No	No	No	Table 1 details teaching activities and units per teaching activity.	This solely focuses on teaching and not on educational scholarship. It also does not take into consideration quality of teaching.	Yes
65: Impact of a financial incentive on the completion of educational metrics, Pugh A, Internatl J Emerg Med, 2020 [22]	Quantitative	Department(s)	Emergency Med (1)	17	Attaching a financial incentive to a tracked educational dashboard increased faculty participation in resident evaluations but did not change conference attendance.	No	Not reported	Completed evals 71.8% to 100%, conference attendance dropped (had to meet quarterly incentive to complete 100% of resident evaluations within 90 days)	No	Not reported	2.5% of annual salary (~$4,500) if minimum achieved Not clear in paper, seems to imply incentive was for complete evals AND attendance; only the former was achieved, but paper implies that $ was allocated	Not reported	Not reported	Not reported	Not reported	Not reported	Not reported	Not reported	Not reported	Not reported	Not reported	Not reported	Not reported	Not reported	Not reported	Not reported	Conference attendance: achieve at least 25% attendance at eligible didactic conference days (deemed eligible if not scheduled for other conflicting clinical or educational activities (eg clinical shift).	Mean conference attendance was similar to post implementation. The 1 faculty member with lowest compliance pre was unchanged after the incentive program.	Yes-ish
1569: Assessment of faculty productivity in academic departments of medicine in the United States: a national survey, Kairouz VF, BMC Medical Education, 2014 [23]	Survey - data handled quant	Department Chairs of Medicine	78 of 152	median 130	Productivity measured: 98% clinical, 61% research, 62% teaching, 64% admin	No	Change not indicated, but use RVUs to measure	Teaching RVUs used to measure (40 respondents) No data re Δ productivity	No	Not reported	Not reported	Not reported	Compensated using incremental amount after min met for non-clinical teaching	Not reported	Not reported	Compensated using incremental amount after min met for clinical teaching	Not reported	Compensated using incremental amount after min met for clinical teaching	Use RVU & grants to measure	Use RVU & grants to measure	Use RVU & grants to measure	Use RVU & grants to measure	Not reported	Not reported	Not reported	Not reported	Seniority, academic rank, career track, dependent on dept performance	No specific improvements in productivity noted, but does describe how medicine departments are compensating	Yes-ish
NEW: The Academic RVU: A system for measuring academic productivity, Mezrich, J ACR, 2007 [24]	Other: Descriptive	Department(s)	Radiology	Not reported	This report describes a productivity-based faculty compensation scheme that can enhance clinical and academic productivity.	No	Not reported	Not reported	No	Not reported	Not reported	Not reported	The following formula is used: aRVUt= % effort * academic value * score (Effort is # hours spent teaching, academic value is the level of learner, score is based on learner evaluation)	The following formula is used: aRVUt= % effort * academic value * score (Effort is # hours spent teaching, academic value is the level of learner, score is based on learner evluation)	Not included as it is considered part of wRVU	Not included as it is considered part of wRVU	Not included as it is considered part of wRVU	Not included as it is considered part of wRVU	Research formula: aRVUr = % effort * academic value * funding modifier * PI status (modifier is based on type of grant - NIH, local, academic value is based on grant dollar range)	Not reported	Research formula: aRVUr = % effort * academic value * funding modifier * PI status (modifier is based on type of grant - NIH, local, academic value is based on grant dollar range)	Not reported	Research formula: aRVUr = % effort * academic value * funding modifier * PI status (modifier is based on type of grant - NIH, local, academic value is based on grant dollar range)	Not reported	Not reported	Not reported	An administrative RVU system was also established to cover committee work.		Yes-ish
NEW: Are the teachers teaching? Measuring the educational activities of clinical faculty, Bardes, Acad Med, 1995 [25]	Quantitative	Description of Relative Value Scale in Teaching for a department of medicine at Cornell.	Internal Medicine	Not reported	Explanation of their point system for teaching activities. They do include administrative points as well.	Not reported	Not reported	Not reported	Not reported	Not reported	Not reported	Not reported	2	2	2	Not reported	2	Not reported	Not reported	Not reported	Not reported	Not reported	Not reported	Not reported	Not reported	Tutor - 1.5	Table 1 provides a breakdown of activities sectioned by GME, UME, ADMIN and SPECIAL PROJECTS	No real data.	Yes-ish
NEW: Quantifying physician teaching productivity using clinical relative value units. Yeh, J Gen Intern Med, 1999 [26]	Quantitative	This report describes a customizable method of quantifying physician teaching productivity in terms of clinical RVUs.	Medicine	11	The authors describe the development of an educational value unit system for a community hospital. The system focuses solely on educational activities and no research.	Consensus process used to agree on the system	Not reported	Not reported	No	Not reported	Not reported	Not reported	1.5	1.5	2	2	1	1	No	No	No	No	No	No	No	No	Table 1 details TVM and hours		Yes-ish
NEW: Development and implementation of a relative value scale for teaching in emergency medicine, Khan, Acad Emer Med, 2003 [27]	Quantitative	This 3-year study reports on the development and progress of an objective measure of teaching productivity linked to a performance-based incentive plan.	Pediatric Emergency Medicine & Pediatrics	18-22	This article reports on the development of their teaching value unit system and numbers collected for years 1-3 of implementation.	The system was developed using a consensus process and approved by the PEM division director	Not reported	Yes, In year 1 only 22% met expectations but by year 3 55% met minimum expectations for teaching.	Not reported	Not reported	A generic model of compensation based on TVU was presented. In yr 3, 12/22 met or exceeded quota and, per protocol, should have been bonused	Although not reported, it was taken into consideration for assigning weight to value unit.	1-2	1-2	1	1	1	1	Not reported	Not reported	Not reported	Not reported	Not reported	Not reported	Not reported	Not	Table 1 provides more of a breakdown for scholarly activities. Interestingly, no credit was given to writing peer-review manuscripts but was awarded for book chapters and journal reviews		Yes-ish
135: Physician-faculty perceptions towards teaching incentives: A case study at a children's hospital, Martinez, Medical Education, 2020 [28]	Quantitative	Children's Hospital	1 (34 clinical divisions)	32/107	Faculty satisfaction with a comp model	Yes	Not reported	Not reported	Not reported	Not reported	A $450 000 allotment was broken into two parts. Part one allocated two-thirds (approximately $300 000) to clinical divisions based on total number of learners who rotated through the division. Part two consisted of two portions: one for educational leadership positions (eg rotation directors) that were not otherwise compensated or given protected time ($60 000; 40%); and another for e-logged academic activity hours ($90 000; 60%). Implementation, distribution of programme details to division chiefs and e-logging began 1 January 2017 and continued through 31 December 2018. The annual teaching bonus pay distribution (N = 107) occurred in May 2018 and 2019 with a mean of $1672.52 (±1821.41).	Not reported	Counted in total time (See Table 1 in manuscript)	Counted in total time (See Table 1 in manuscript)	Counted in total time (See Table 1 in manuscript)	Counted in total time (See Table 1 in manuscript)	Counted in total time (See Table 1 in manuscript)	Counted in total time (See Table 1 in manuscript)	N/A	N/A	N/A	N/A	N/A	N/A	N/A	Counted in total time (See Table 1 in manuscript)		Table 1 addresses aspects of educational service and teaching that are given credit to receive the incentive. hard to know if only the highlighted activities were counted. Median bonus: $1672.52 (±1821.41)	Yes, but limited
214: An Academic Relative Value Unit System, Carmody, West J Emerg Med, 2019 [29]	Quantitative	Other: EM	One	123	Increase educational 'productivity' and realignment of effort across faculty based on other work (such as clinical 'load')	NR - but implication is that it was measured	no	yes-attendance and participation in teaching and completion of resident performance assessments	No	No	Yes- built into incentive plan (tiered system- minimal expectations first needed to be met and then aRVU applied: voluntary) Specific amounts not yet known when paper published.	no	Yes	can't tell	Yes (some- first night call for interns, tox fellow rounds). Does not appear to be value for most clinical work	No	NA	NA	Yes	Yes	Yes	Yes	Yes	Yes	Yes	Yes	Committee/leadership work, applicant interviews etc (Table 2)	NA	Yes, but. This is a mix of 3 items used to track faculty AND the development, but not implementation, of a really granular aRVU system. Columns M through AB are (mostly) included, with points assigned, but for the year of the study, these were tracked - not clearly counted for incentive/compensation.
505: Using Organizational Philosophy to Create a Self-Sustaining Compensation Plan Without Harming Academic Missions, Leverence R, Acad Med, 2017 [30]	Quant + Survey	Department(s)	Medicine	Not reported	The new compensation plan was implemented in FY2013. A survey administered at the end of FY2015 showed that 61% (76/125) of faculty were more satisfied with this plan than the previous plan. Since the year before implementation, clinical relative value units per faculty increased 7% (from 3,458 in FY2012 to 3,704 in FY2015, P < .002), incentives paid per faculty increased 250% (from $3,191 in FY2012 to $11,153 in FY2015, Pâ‰¤.001), and publications per faculty increased 15% (from 2.6 in FY2012 to 3.0 in FY2015, P < .001). Grant submissions, external funding, and teaching hours also increased per faculty but did not reach statistical significance.	Yes Generally +	Increased RVUs by 7%; incentives increased by 250%	Teaching hours increased per faculty	No	Bottom up program - there was increased revenue to more to depts State general revenue for medical education is disbursed by the college based on face time with medical students prorated for the number of students involved. Thus, more medical student teaching effort resulted in more revenue to the department.	Average incentives earned per faculty increased 250% since the year before implementation, from $3,191 in FY2012 to $11,153 in FY2015 Faculty at < 75% of AAMC salary benchmarks could take half of the prior yearâ€™s incentive as a base salary increase, with their RVU targets then concurrently increased. This meant that a choice to increase salary led to increased responsibility for productivity as well. Thus, salaries generated by the plan were both self-funding and self-correcting.	Not reported	Not reported	Not reported	Not reported	Not reported	Not reported	Not reported	Not reported	Not reported	Not reported	Not reported	Not reported	Not reported	Not reported	Not reported	The RVU benchmark for a given salary and rank was set at 1, and then the fractional clinical effort was subtracted from that, leaving a fraction of RVUs that needed to be covered by educational or research effort. Educational and research productivity was measured by revenue generated from educational or research effort. If it covered that fraction of salary then it met the RVU target. If the revenue generated exceeded the target, it was converted into RVUs by dividing the excess funding by the total salary, multiplied by the total RVU target. All education or research revenue (such as state medical education funds given for teaching medical students, graduate medical education funds for postgraduate training, or external research grants or contracts) that was generated by an individual faculty was translated into RVUs, based on the average dollar per clinical RVU. The total RVUs generated by an individual facultyâ€™s combined efforts were set by cross-matching AAMC percentile salary with UHC percentile RVUs. Some educational efforts, notably fellowship and residency administration, and most start-up and bridge research efforts, had to be internally funded, usually from foundation funds, such as the Gatorade Trust, or residual clinical revenue from the prior year. These were translated into RVUs and counted toward the annual target. However, only additional external funds could result in an incentive for that faculty.		Yes, in a way. Time-based: "For the TFTE, actual hours as recorded by the DoM and CoM will be used for calculating the actual TFTE, based on 46 annual productive weeks and 60 hours per week, and converted to the actual RVUs." There was punishment for professionalism issues - individual or division. "Quality of teaching was rewarded outside of the compensation plan"
496: The Academic RVU: Ten Years Developing a Metric for and Financially Incenting Academic Productivity at Oregon Health & Science University, Ma, Academic Medicine, 2017 [31]	Quantitative	Department(s)	Emergency Medicine	42 in most recent academic year: 31 practicing faculty clinicians, 3 nonclinical research faculty, and 8 fellows. The median number over the 10-year period was 43 .	Points earned and amount of academic bonus	No	Not measured - one can infer it did not drop based on comments re incentive pool	Paper separates Educational from Scholarly productivity. Clearly the latter increased. Although (see pg 9 of appendix), there was not a steady upward slope of bonus $	No	No	Yes The total annual financial bonus pool ranged from $211,622 to $274,706. The median annual per faculty academic bonus remained fairly constant over time ($3,980 in 2005-2006 vs. $4,293 in 2014-2015), with most change at the upper quartile of academic bonus (max bonus $16,920 in 2005-2006 vs. $39,207 in 2014-2015). Bonuses rose linearly among faculty in the bottom three quartiles of academic productivity, but increased exponentially in the 75th to 100th percentile	NR	Core lecture to EM residents including intern lectures: 4 points per hour of lecture a. Additional weight is given to our core lecture series to stimulate faculty member participation. b. All faculty members are expected to provide at least 2 hours of didactic teaching each year in the residency core curriculum Lecture to other OHSU residents, medical students, paramedics, paramedic students, etc: 1 point for each hour. Lecture at regional/national meetings: 2 points per hour PCM tutoring: 1 point per hour of class room tutoring/lecturing;	NR	Not included	PCM tutoring: 1 point per 20 hours of preceptorship IN THE EM SETTING (not really hospital team)	Not included	PCM tutoring: 1 point per 20 hours of preceptorship IN THE EM SETTING (not really hospital team)	Complex system - see appendix	Complex system - see appendix	Complex system - see appendix	Complex system - see appendix	Included in scholarly product category, Complex system - see appendix	Included in scholarly product category, Complex system - see appendix	Included in scholarly product category, Complex system - see appendix	Not included	For the education category: also participation in formal School of Medicine education activities, organizing Journal Club, directing School of Medicine courses, serving on national or regional committees with an educational focus (see appendix for values)	Data for values from 2014-2015 taken from appendix found at http://links.lww.com/ACADMED/A422 They accumulate points based on their activity (numerator), and the cumulative points of all faculty members serve as the denominator The dollar value of a "point" has varied over time according to the total funding allocated to the academic bonus pool, the number of participating faculty, total number of faculty academic activities, and changes to the academic bonus point system. However, within a given academic year, the value of a point is constant among all faculty members, allowing financial compensation based on standardized academic productivity for each faculty member.	Yes. Note: "Finally, we are unable to test a causal relationship with these data. The data do not allow us to assess whether implementation and use of the academic bonus system incented greater academic productivity and engagement than would have occurred without the system."
878: Implementation of an Education Value Unit (EVU) System to Recognize FacultyContributions, House, Western Journal of Emergency Medicine, 2015 [32]	Quantitative	Other: Emergency Medicine	1	50	1) determine the feasibility of an EVU system in an academic ED 2) to examine its effect on faculty behavior in the educational mission	No	N/A	Yes - across board - specifics in Table 2	No	N/A	Yes, the 1% was distributed Max bonus 1%	N/A	10 hours/hour	5 hours/hour	No	No	No	No	No - teaching-specific metrics	No - teaching-specific metrics	No - teaching-specific metrics	No - teaching-specific metrics	No - teaching-specific metrics	No - teaching-specific metrics	No	1 hour/hour	Attending conferences, evaluating residents/fellows, interviews, activities outside department, committees	EVU system was "one component of an eight-part overall ED incentive system representing educational, scholarly, and clinical goals each with an equal weight applied universally to all faculty."	Yes. Not a lot of detail, but enough. EVUs were measured in hours. I cannot see if/how they converted an hour to an RVU-comparable metric.
Measuring Faculty Effort: A Quantitative Approach That Aligns Personal and Approach That Aligns Personal and Institutional Goals in Pathology at Yale. Morrow 2021 [33]	Quantitative	Department	One Pathology	62	Unifying metric	"Well-received" How this was determined is not measured.	N/A	N/A	No	N/A	N/A	N/A	0.1 for lecture, 1.0 [week effort] for new lecture prep	0.1 week effort	N/A	N/A	N/A	N/A	Based on funds	Complex system - see text	Based on funds	Complex system - see text	Based on funds	Complex system - see text	0.1-1 week effort	N/A	Organizing nat'l/int'l meeting	Developed SWAY software to analyze inputs	Yes
Mission Aligned Management and Allocation: A Successfully Implemented Model of Mission-based Budgeting Ridley 2002 [6]	Quantitative/Descriptive	Design & Implementation of school-wide MBM	Not reported	NR	Implementation	No	NR	"increased academic productivity"	No	NR	NR	NR	NR	NR	NR	NR	NR	NR	NR	NR	NR	NR	NR	NR	NR	NR	NR	2nd yr of implementation. See list 2 for criteria that were included - not granular	A little
Aligning Compensation with Education: Design and Implementation of the Educational Value Unit (EVU) System in an Academic Internal Medicine Department Stites 2005 [34]	Quantitative	Department	1 - Int Med	57	Implementation	Yes - better reflected education effort	NR	NR - except "dramatic improvement in attendance"	No	Increased	37/54 had a DECREASE in university salary support, offset by incr clinical vols	NR	NR	NR	0.02 EVU/month - any learner	#NAME?	0.002 EVU/half-day cont clinic	0.001 EVU/half day	NR	NR	NR	NR	NR	NR	NR	NR	Consults with Learners - 0.015 EVU/month	0.1 EVU = $12,562 - core expectation: 0.2 EVU/yr	Yes
Adapting Industry-Style Business Model to Academia in a System of Performance-Based Incentive Compensation Reese 2008 [35]	Quantitative	School-wide	21	676	Implementation	Yes - "improved" not clearly reported - see figure 6	Increased 5-12% each year	NR	No	Increased	Increased - avg 20%	NR	NR	NR	NR	NR	NR	NR	NR	NR	NR	NR	NR	NR	NR	NR	NR	See text #3: "Academic performance is determined using…"	Yes
Measuring Physicians’ Productivity - A 3-yr Study of a New Remuneration System Filler 2014 [36]	Quantitative	Department	1 - Pediatrics	37	Implementation	No	No - except for Asst professors	NR	NR	NR	No - except perhaps for Asst Prof's	NR	NR	NR	NR	NR	NR	NR	NR	NR	NR	NR	NR	NR	NR	NR	NR	Maybe incentives should be 20-30% See Appendix 1 for scoring rubric	Maybe
Implementing a Simpler Approach to Mission-Based Planning in a Medical School Sloan 2005 [37]	Quantitative	School-wide	13	893	Implementation	No	NR	NR	No	NR	NR	NR	10.4 hrs new 3.7 hrs substantial update	1.3 hrs	Not clear	50% of time spent	NR	50% of time spent	Yes	NR	Yes	NR	NR	NR	NR	NR	NR	80% of state-appropriated revenue to the teaching mission and 20% to administrative activities. Allocated salary expenses based on % of faculty time spent in each of the 4 missions. No effort was made to assign relative values to educational, clinical, research, or scholarly activities - time only	Yes
The Impact of a Program for Systematically Recognizing and Rewarding Academic Performance Williams 2003 [38]	Mixed	Department	1 - Surgery	33 - 20 analyzed	Implementation/Evaluation	Yes: 26% very, 44% somewhat satisfied	NR - implied that it did not change	Trend decreased over study, BUT more faculty got incentive $	No	No	NR	NR	NR	NR	22 credits	22 credits - unclear of residents or students were differentiated	NR	NR	NR	22 credits	NR	22 credits	NR	22 credits	NR	NR	22 credits for organizing and delivering a CME conference	Incentive = 1% of the practice plan’s income. No credit for teaching activities in conjunction with pt care nor for planning (a lecture) Unit of measure was a "credit"	Yes
A Mission-Based Reporting System Applied to an Academic Pathology Department Howell Human Path 2003 [39]	Quantitative	Department - but part of UC Davis roll out	1 - Pathology	29	Implementation	NR	NR	NR	NR	NR	NR	NR	NR	NR	NR	NR	NR	NR	NR	NR	NR	NR	NR	NR	NR	NR	NR	50-hr work week = 1 FTE. Targeted School Avg: 32% rsch, 25% tch, 32% clin, 11% admin Path: 50% rsch, 50% tch, 17% admin, 38% clin = 155%	Yes, but limited
Mission-Based Reporting in Academic Psychiatry Anders 2004 [40]	Quantitative	Department - but part of UC Davis roll out	1 - Psychiatry	35	Implementation	NR	NR	NR	NR	NR	NR	NR	NR	NR	NR	NR	NR	NR	NR	NR	NR	NR	NR	NR	NR	NR	NR	50-hr work week = 1 FTE. Targeted School Avg: 32% rsch, 25% tch, 32% clin, 11% admin Psych: 48% rsch, 35% tch, 18% admin, 25% clin = 126%	Yes, but limited
A Progress Report on a Department of Psychiatry Faculty Practice Plan Designed to Reward Educational and Research Productivity Hales 2009 [41]	Quantitative	Department - but part of UC Davis roll out	1 - Psychiatry	41 - 66% turnover	Progress report	NR	NR	NR	NR	NR	NR	NR - but incr papers and grants	NR	NR	NR	NR	NR	NR	NR	4% of base salary	NR	4% of base salary	NR	4% of base salary	NR	50% of contact hours	See Table 2	9 years and still no quality component	Yes, but limited
